# The Heat Shock Response in the Western Honey Bee (*Apis mellifera*) is Antiviral

**DOI:** 10.3390/v12020245

**Published:** 2020-02-22

**Authors:** Alexander J. McMenamin, Katie F. Daughenbaugh, Michelle L. Flenniken

**Affiliations:** 1 Department of Plant Sciences and Plant Pathology, Montana State University, Bozeman, MT 59717, USA; alexmcmenamin2@gmail.com (A.J.M.); kdaughenbaugh@gmail.com (K.F.D.); 2 Department of Microbiology and Immunology, Montana State University, Bozeman, MT 59717, USA; 3 Pollinator Health Center, Montana State University, Bozeman, MT 59717, USA

**Keywords:** honey bee, *Apis mellifera*, heat shock, thermal stress, antiviral immunity, RNA virus, insect virus, insect antiviral defense

## Abstract

Honey bees (*Apis mellifera*) are an agriculturally important pollinator species that live in easily managed social groups (i.e., colonies). Unfortunately, annual losses of honey bee colonies in many parts of the world have reached unsustainable levels. Multiple abiotic and biotic stressors, including viruses, are associated with individual honey bee and colony mortality. Honey bees have evolved several antiviral defense mechanisms including conserved immune pathways (e.g., Toll, Imd, JAK/STAT) and dsRNA-triggered responses including RNA interference and a non-sequence specific dsRNA-mediated response. In addition, transcriptome analyses of virus-infected honey bees implicate an antiviral role of stress response pathways, including the heat shock response. Herein, we demonstrate that the heat shock response is antiviral in honey bees. Specifically, heat-shocked honey bees (i.e., 42 °C for 4 h) had reduced levels of the model virus, Sindbis-GFP, compared with bees maintained at a constant temperature. Virus-infection and/or heat shock resulted in differential expression of six heat shock protein encoding genes and three immune genes, many of which are positively correlated. The heat shock protein encoding and immune gene transcriptional responses observed in virus-infected bees were not completely recapitulated by administration of double stranded RNA (dsRNA), a virus-associated molecular pattern, indicating that additional virus–host interactions are involved in triggering antiviral stress response pathways.

## 1. Introduction

Honey bees (*Apis mellifera*) are eusocial insects in the order Hymenoptera that live in colonies consisting of one reproductive queen, hundreds of reproductive males (drones), and approximately 30,000 non-reproductive sterile female workers. Honey bees are generalist pollinators of numerous plant species, including fruit, nut, and vegetable crops [1,2,3] valued at $14.6 billion annually in the United States [4]. Unfortunately, the U.S. and parts of Europe have experienced unsustainable annual losses of honey bee colonies (e.g., 33% average yearly losses since 2006 in USA) [5,6,7,8,9,10,11]. Colony losses are attributed to numerous biotic and abiotic stressors, including inadequate nutrition, the *Varroa destructor* mite, and pathogens [12,13]. There are numerous viruses that infect honey bees and the prevalence and abundance of several of them, including deformed wing virus, have been associated with colony losses globally [10,11,14,15,16,17,18,19,20,21,22,23,24,25,26].

Positive-sense single-stranded RNA viruses comprise the largest group of known honey bee pathogens (reviewed in [27,28]). These include members of *Iflaviridae* (i.e., deformed wing virus, sacbrood virus, and slow bee paralysis virus), *Dicistroviridae* (i.e., black queen cell virus, Israeli acute paralysis virus, acute bee paralysis virus, and Kashmir bee virus), chronic bee paralysis virus [29], and Lake Sinai viruses [15,30,31], as well as a growing list of other viruses and virus families (reviewed in [27,28,32]). Viruses are transmitted vertically from parents to offspring and horizontally between bees within the crowded environment of the colony via contact and trophallaxis (mouth to mouth food transfer), as well as via contact with contaminated floral resources while foraging [33,34] (reviewed in [28,35]). In addition, several viruses are transmitted by the ectoparasitic mite, *Varroa destructor* (reviewed in [22,27,28]). Like other organisms, honey bees have evolved sophisticated immune systems to limit virus infections [36] (reviewed in [37,38]). Unlike many organisms, honey bees also have social immune mechanisms [39,40] (reviewed in [41,42,43]). Although it is unclear how social immunity affects viral infections, hygienic behaviors that reduce mite infestation likely reduce mite-mediated virus transmission (reviewed in [41,43]).

Antiviral defense at the individual bee level impacts the health of the entire colony. Honey bee colonies are considered superorganisms and, therefore, require a sufficient number of workers to keep the colony warm, feed the developing brood, and forage. Honey bee antiviral defense mechanisms include autophagy, endocytosis, apoptosis, eicosanoid signaling, melanization, and the Toll and Imd (Immune Deficiency) pathways involving NF-κB (Nuclear Factor κB) homologues Dorsal and Relish, JAK/STAT (Janus Kinase/Signal Transducer and Activator of Transcription), JNK (c-Jun N-terminal kinase), MAPK (Mitogen-Activated Protein Kinases), and RNA interference (RNAi) pathways [14,20,44,45,46,47,48,49,50,51]. RNAi is a sequence-specific post-transcriptional gene silencing mechanism. The short-interfering RNA (siRNA) directed RNAi pathway is the primary insect antiviral response, and is induced upon recognition of virus-derived double stranded RNA (dsRNA) [37,44,45,51,52,53,54,55,56,57,58,59,60]. In honey bees and bumble bees, administration of dsRNA, a virus-associated molecular pattern (VAMP), results in a non-sequence specific antiviral response and impacts gene expression [46,61,62,63,64,65,66].

Transcriptome analyses of honey bees identified hundreds of differentially expressed genes in response to dsRNA [46,61,66] and virus infection including RNAi pathway members and genes encoding antimicrobial peptides and heat shock proteins (HSPs) [14,48,49,61,67]. A recombinant Sindbis virus that expresses green fluorescent protein (SINV-GFP) has been used to investigate antiviral defense in a wide range of insects including fruit flies [68], mosquitos [69], and honey bees [46,61]. SINV-GFP is easily produced in cultured cells, trackable, and does not encode an RNAi suppressor protein, and thus facilitates investigation of this and other insect antiviral response pathways. Honey bees infected with SINV-GFP exhibited increased expression of *ago-2*, *dcr-like*, *mf116383*, and numerous heat shock stress response pathway members including *heat shock protein 90, activator of hsp90, hsp60, heat shock protein 83-like, dnaj shv-like, protein lethal(2)essential for life-like,* and *hsp10* [61]. Whereas *heat shock protein 70 cognate 3* expression was increased in bees naturally infected with Israeli acute paralysis virus (IAPV) in one study, its expression was decreased in bees experimentally inoculated with IAPV in a second study [14,48]. Additional experiments are needed to elucidate the role of the heat shock stress response pathway in honey bee antiviral defense.

In general, heat shock proteins (HSPs) are involved in maintaining cellular protein homeostasis—protein synthesis, folding, function, and regulation of degradation (i.e., proteostasis) [70]. The heat shock response and broader proteostasis network in honey bees are conserved and the expression of core heat shock factor target genes and several members of the broader proteostasis network are induced by heat-stress [71]. There is a positive relationship between the heat shock response and immune response in other invertebrates, including *Drosophila melanogaster* and *Caenorhabditis elegans* [72,73,74,75,76]. However, McKinstry et al. determined that heat shock factor target gene expression was reduced in response to wounding and bacterial infection in honey bees and thus concluded that the heat shock response and humoral immune responses are mutually antagonistic [71]. In the context of viral infection, HSPs can both positively and negatively affect viral replication [76,77,78,79,80,81]. For example, heat shock protein 90 (Hsp90) facilitates the assembly of Flock House virus (FHV) replicase in *Drosophila* S2 cells, while heat shock protein 40 (Hsp40) and Hsp70 family members have contrasting effects on FHV replication in yeast [79,80]. Additionally, HSPs are required for assembly of the RNAi-induced silencing complex (RISC) and effective RNAi defense against viruses in *Drosophila melanogaster* [77,78]. Intriguingly, heat-stress (temperatures >37 °C) reduced deformed wing virus (DWV) loads in naturally infected honey bees [82,83].

Therefore, given the transcriptional regulation of HSP-encoding genes in virus-infected honey bees, the role of HSPs in insect RNAi, and the experimental reduction of naturally occurring DWV infections in heat-stressed honey bees, we hypothesized that HSPs and the heat shock response are involved in honey bee antiviral defense [14,48,49,61,77,78,82,83]. To test this hypothesis, we examined the effect of heat shock (42 °C for 4 h) on honey bees experimentally infected with a model virus SINV-GFP, via intrathoracic injection, an infection route that mimics mite-mediated virus transmission and ensures precise delivery of a known dose. The relative expression of HSPs and immune genes was assessed by quantitative PCR (qPCR). We determined that both viral infection and heat shock induced the expression of HSPs, though expression in bees exposed to heat shock alone varied. The relative expression of HSPs and immune genes was positively correlated, suggesting potential co-regulation of these genes in response to stressors. Lastly, double stranded RNA, which is a hallmark of virus infection, was not sufficient to recapitulate the HSP induction pattern observed in virus-infected honey bees. This indicates that virus–host interactions beyond molecular pattern recognition are required to induce the expression of some HSP-encoding genes in honey bees.

## 2. Materials and Methods

### 2.1. Honey Bees

Honey bee (*Apis mellifera)* colonies were established from packages (~1.5 kg of worker bees and a naturally-mated queen) of primarily *Apis mellifera carnica* stock purchased from a commercial producer in Montana in April 2018. Honey bees were kept in Langstroth hives located on Montana State University’s Horticulture Farm in Bozeman, MT, USA. Colonies were maintained using standard apicultural practices, including bi-monthly evaluation of *Varroa destructor* mite infestation levels using the powdered sugar roll method [84]. Colonies were treated with formic acid polysaccharide gel strips (Mite Away Quick Strips^®^, Nature’s Own Design Apiary Products, Frankford, ON, Canada) when mite infestation was greater than 3% (3 mites per 100 bees) [84,85].

Honey bees for laboratory-based experiments were obtained from frames of newly emerging bees, which were collected one day prior to each experiment and maintained at 32 °C in a laboratory incubator overnight. Young, age-matched (~24 h post-emergence), female adult bees were utilized for all experiments. For the duration of the experiment, honey bees were housed in modified deli-containers at 32 °C and fed bee candy (powdered sugar mixed with corn syrup until pliable) and water *ad libitum* [46,86].

### 2.2. dsRNA Preparation

Double stranded RNA was generated by in vitro transcription with T7 RNA polymerase [61,68]. T7 promoter-containing PCR-products were amplified using primers listed in Table S1, with the following thermocycler program: pre-incubation of 95 °C (5 min), 35 cycles of 95 °C (30 s), 60 °C (30 s), and 72 °C (1 min) followed by a final elongation at 72 °C (5 min). PCR products were used as template for T7 polymerase transcription (100 μL reactions: NTPs (each 7.5 mM final), RNase OUT (40 units) (Invitrogen, Carlsbad, CA, USA), buffer (400 mM HEPES pH 7.5, 120 mM MgCl_2_, 10 mM spermidine, 200 mM DTT); reactions were carried out at 37 °C overnight (8–10 h). DNA was removed by adding 1 unit of RQ1 DNAse (Promega, Madison, WI, USA) and incubating for 15 min at 37 °C. dsRNA products were precipitated with 500 μL ethanol and 1:10 volume 3 M sodium acetate (pH 5.2), suspended in 200 μL RNase-free water, and annealed at 100 °C for 5 min and then slowly cooled to room temperature. dsRNA products were purified by phenol:chloroform extraction and subsequent precipitation with 600 μL ethanol and 80 μL 5 M ammonium acetate. RNA pellets were dissolved in 60–100 μL 10 mM Tris HCl (pH 7.5). Quality was assessed by agarose gel electrophoresis and spectrophotometry. The dsRNA quantity based on gel band intensity relative to a standard was determined using ImageJ version 1.50i [87].

### 2.3. Virus Infection and Heat Shock Protocol

Glass needles for honey bee intra-thoracic injections were made by pulling borosilicate glass capillary tubes (100 mm long, 1 mL capacity, Kimble-Chase) with a coil temperature of 61 °C on the PC-10 Dual-Stage Glass Micropipette Puller (Narishige, East Meadow, NY, USA). Prior to injection, age-matched honey bees (~24 h post-emergence) were cold anesthetized for 10 min at 4 °C. Honey bees were infected with SINV-GFP (3750 plaque forming units (PFU) in 2 μL 10 mM Tris HCl buffer pH 7.5) via intra-thoracic injection using a Harbo syringe (Honey bee Insemination Service) and microcapillary glass needles; mock-infected bees were injected with 2 μL buffer (10 mM Tris HCl, pH 7.5) [46,61]. The dose of 3750 PFUs SINV-GFP per honey bee was based on our previous studies that determined that this dose allowed for a natural progression of infection over the course of the experiment [46]. By 72 h post-infection virus could be visualized by fluorescence microscopy and relatively quantified by Western blot analysis and qPCR [46,61]. Furthermore by 72 h post-infection. changes in gene expression reflected honey bee antiviral defense mechanisms [46,61]. This dose is modest compared to drosophila studies which typically utilize 250–2500 PFUs per fly; a newly emerged female worker honey bee (~150 mg) weighs ~200× more than an adult female fruit fly (0.8 mg) [68].

Experimental treatment groups that were subjected to temperature stress were intrathoracically injected with either buffer or virus, allowed to recover for 6 h at 32 °C (i.e., ample time for post-injection recovery, but less than the amount of time estimated for a virus replication cycle), exposed to heat shock (i.e., 42 °C for 4 h) [88], and then transferred back to 32 °C for the remainder of the study. The heat shock experiments were carried out using three independent honey bee cohorts obtained from three different colonies on distinct dates (i.e., replicate 1 in June 2018, replicate 2 in August 2018, and replicate 3 in July 2019).

### 2.4. RNA Isolation

Honey bee samples were dissected into head, thorax, and abdomen. The abdomen was chosen for further analysis as it is the primary site of immune cell-generating fat bodies and it is distal from the site of injection, and thus virus infection naturally spread to that tissue. Honey bee abdomens were homogenized in 300 μL of deionized water with a sterile steel ball (5 mm) using a Tissue Lyser II (Qiagen, Germantown, MA, USA) at 30 Hz for 2 min. Then, 300 μL of TRIzol reagent (Invitrogen) was added to the homogenate, vortexed for 15 s and incubated at room temperature for 5 min. Next, 100 μL of chloroform was added, samples were shaken by hand for 15 s and incubated on the benchtop for another 2 min. Samples were then centrifuged at 12,000*× g* at 4 °C for 15 min and the aqueous phase was transferred to a clean centrifuge tube. One volume of isopropanol was added to the aqueous phase, mixed by inversion, and nucleic acid was precipitated by incubation at room temperature for 10 min. The precipitate was pelleted by centrifugation at 12,000× *g* at 4 °C for 10 min. Pellets were then washed with 500 μL of 75% ethanol and centrifuged at 7500× *g* at 4 °C for 5 min, then air dried for 10 min at room temperature and dissolved in 30 μL of deionized water. RNA concentrations and quality were assessed on a Nanodrop 2000 spectrophotometer (Thermo Fisher). When quality was low, RNA was precipitated a second time by addition of 4 volumes of cold ethanol and 1:10 of a volume 3 M sodium acetate (pH 5.5) and incubation at −20 °C overnight. Nucleic acids were pelleted by centrifugation at 12,000× *g* at 4 °C for 10 min and pellets were washed one time with 500 μL 70% ethanol and centrifuged at 12,000× *g* at 4 °C for 5 min. Pellets were air dried and suspended in 30 μL deionized H_2_O. Samples were stored at −80 °C until analysis.

### 2.5. Reverse Transcription/cDNA Synthesis

Reverse transcription reactions were performed by incubating 2000 ng total RNA, 200 units M-MLV reverse-transcriptase (Promega) and 500 ng random hexamer primers (IDT) for 1 h at 37 °C, according to the manufacturer’s instructions. cDNA was diluted 1:2 and 2 μL was used for PCR or qPCR analysis.

### 2.6. Polymerase Chain Reaction (PCR)

Polymerase chain reaction (PCR) was used to test replicate experiments for pre-existing infections with honey bee infecting viruses since bees were obtained from honey bee colonies which are subject to naturally occurring infections. Pools were made from the cDNA of 4 bees (*n* = 8–12 bees total), that received no injection but were age-matched to the experimental bees, which were tested for pre-existing/confounding infections by pathogen-specific PCR (Figure S3). Honey bees were tested for black queen cell virus (BQCV), chronic bee paralysis virus (CBPV), acute bee paralysis virus (ABPV), deformed wing virus (DWV), Israeli acute bee paralysis virus (IAPV), Kashmir bee virus (KBV), sacbrood virus (SBV) and Lake Sinai viruses (LSVs 1–4). PCR was performed according to standard methods [89]. In brief, 2 μL cDNA template was combined with 10 pmol of each forward and reverse primer (Table S1), and amplified with Denville ChoiceTaq polymerase (Thomas Scientific, Swedesboro, NJ, USA) according to the manufacturer’s instructions using the following cycling conditions: 95 °C for 5 min, 95 °C for 30 s, 57 °C for 30 s, 72 °C for 30s, 35 cycles, followed by final elongation at 72 °C for 4 min.

### 2.7. Quantitative PCR (qPCR)

Quantitative PCR was used to analyze the abundance of SINV-GFP and the relative abundances of honey bee transcripts expressed by immune and heat shock protein encoding genes. All qPCR reactions were performed in triplicate with 2 μL of cDNA template. Each 20 μL reaction contained 1× Denville ChoiceTaq Mastermix, 0.4 μM each forward and reverse primer, 1× SYBR Green (Life Technologies, Carlsbad, CA, USA), and 3 mM MgCl_2_. A CFX Connect Real Time instrument (BioRad, Hercules, CA, USA) was used for the following thermo-profile: pre-incubation 95 °C for one minute followed by 40 cycles of 95 °C for 10 s, 58 °C for 20 s, and 72 °C for 15 s, with a final melt curve analysis at 65 °C for 5 s to 95 °C.

**Figure 1:**
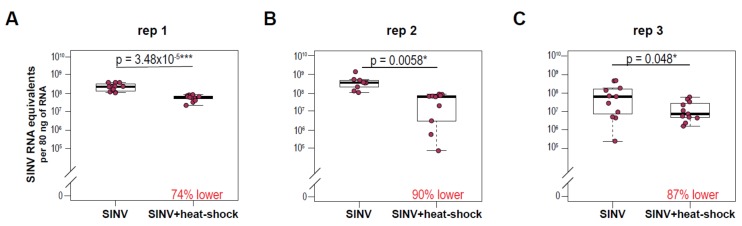
Heat shock reduces virus abundance in adult honey bees. Virus (SINV-GFP) abundance in individual honey bees at 72 h post-infection was assessed by qPCR. Data from three biological replicates with honey bees (*n* = 9–12) obtained from different colonies (rep 1, 2, 3) illustrate that bees subjected to heat shock (42 °C for 4 h) following SINV-GFP injection harbored less virus than bees maintained at 32 °C for the duration of the experiment. (**A**) Replicate 1, SINV-infected bees maintained at 32 °C had a mean viral abundance of 2.1 × 10^8^ ± 2.8 × 10^7^ (*n* = 12) SINV RNA copies, compared to 5.44 × 10^7^ ± 6.72 × 10^6^ (*n* = 11) SINV RNA copies in heat-shocked bees (ANOVA, F = 27.16, *p* = 3.48 × 10^−5^), (**B**) Replicate 2, SINV-infected bees maintained at 28°C had a mean viral abundance of 4.28 × 10^8^ ± 1.28 × 10^8^ (*n* = 9) compared to the 90% lower mean virus abundance in heat-shocked bees of 4.9 × 10^7^ ± 1.1 × 10^7^ (*n* = 9) SINV RNA copies (ANOVA, F = 9.93, *p* = 0.0058) and (**C**) Replicate 3, SINV injected bees had a mean viral abundance of 1.32 × 10^8^. Significance levels: * *p* < 0.05; ** *p* < 0.005; *** *p* < 0.0005.

**Figure 2:**
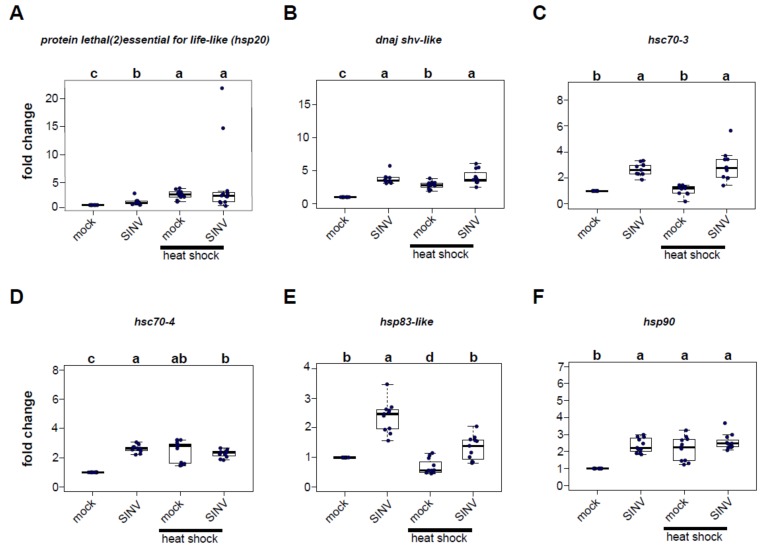
Induction of heat shock genes by virus infection and heat shock. The relative expression of honey bee heat shock protein encoding genes was assessed by qPCR using the ΔΔ*C*t method with normalization to *rpl8* and relative to mock-infected bees maintained at a constant temperature, unless otherwise stated. (**A**) *Protein lethal(2)essential for life-like (hsp20)* expression is increased by both viral infection and heat shock, including when stressors are combined. (**B**) *Dnaj shv-like* is increased in expression by both viral infection and heat shock, including when stressors are combined. (**C**) *Heat shock 70-kDa protein cognate 3 (hsc70-3)* expression was induced by viral infection, and in bees subjected to both stressors, but not by heat shock alone. (**D**) *Heat shock 70-kDa protein cognate 4 (hsc70-4)* expression was increased in all three treatment groups (i.e., virus infection, heat shock, and combined stressors). (**E**) *Heat shock protein 83-like* (*hsp83-like)* expression was induced by virus infection, while heat shock reduced expression in two out of three biological replicates. The combination of virus infection and heat shock also reduced *hsp83-like* expression relative to viral infection alone in all three replicates, indicating heat shock may suppress expression. (**F**) *Heat shock protein 90 (hsp90)* expression was increased in virus-infected bees, heat-shocked mock-infected bees, and by the combination of both stressors. Data were analyzed by a pairwise Wilcoxon Rank Sums with a Benjamini–Hochberg correction for multiple comparisons within replicates (*n* = 9–12 bees for each sample group). Shared letters above two treatments denote there is no difference while different letters denote a statistical difference. Figure 2 includes results from a representative biological replicate for each gene (i.e., rep1 for panel **A**, and rep3 for **B**–**F**). The data for all three biological replicates are presented in Figure S1.

**Figure 3:**
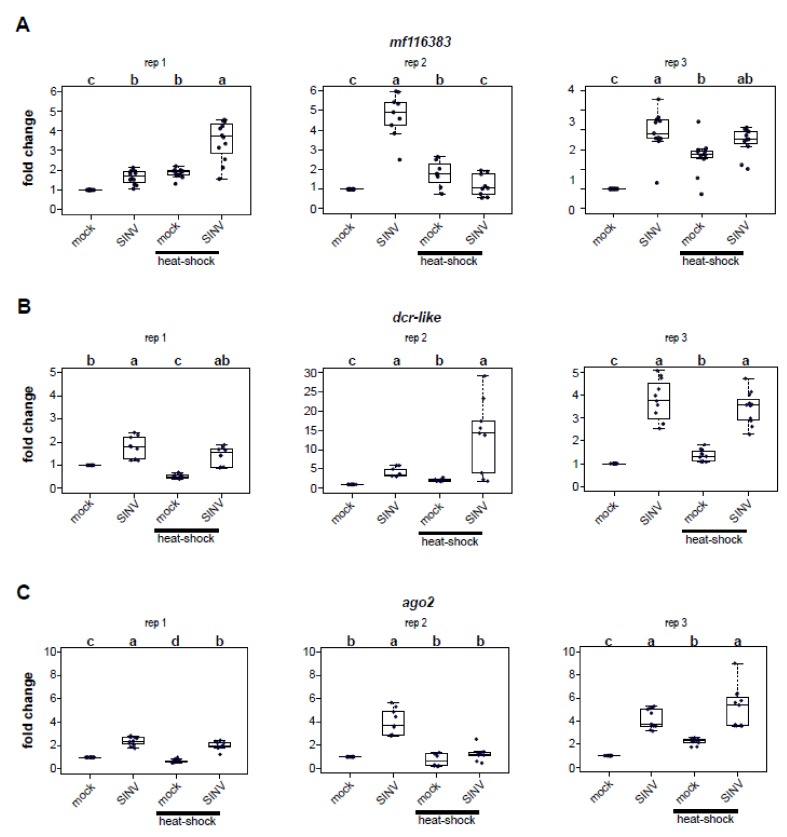
Effect of virus infection and heat shock on honey bee antiviral gene expression. The relative expression of three honey bee antiviral genes was assessed using qPCR using the ΔΔ*C*t method with normalization to *rpl8* and relative to expression in mock-infected bees kept at constant temperature. (**A**) *Dicer (dcr)-like* expression in virus-infected honey bees was consistently increased, while heat shock resulted in decreased expression in replicate 1 and increased expression in replicates 2 and 3. (**B**) Similarly, *ago2* expression was consistently increased in virus-infected bees relative to mock-infected bees, but heat shock resulted in a variable response. (**C**) *Mf116383* expression was consistently induced in virus-infected bees relative to those that were mock-infected. *Mf116383* was the only honey bee antiviral gene that exhibited increased expression in mock-infected heat shocked bees relative to mock-infected bees kept at a constant temperature in all three replicates (*n* = 9–12 for each group). All pairwise comparisons were analyzed by a Wilcoxon rank sums test with a Benjamini–Hochberg correction for multiple comparisons. Shared letters above two treatments denote no difference while different letters denote a statistical difference, and *p*-values for specific comparisons are reported in the text.

To quantify viral genome copy numbers in the samples, SINV-GFP plasmid standards were used as templates, with concentrations ranging from 10^3^ to 10^9^ copies per reaction to create a linear standard curve. The detection limit was 10^3^ copies of the SINV cDNA using primers qSindbisFW4495 and qSindbisREV4635. The host gene *Am rpl8* was amplified in triplicate for each sample for comparison, using primers Rpl8-Fw1 and Rpl8-Rev1 (Table S1). Reactions without template were carried out as negative controls. qPCR specificity was verified through melt point analysis and via gel electrophoresis, and all products had previously been verified by sequencing. The linear equation for the plasmid standard for SINV was: *C*t = −3.348*x* + 40.25 (*R*^2^ = 0.996, efficiency = 98.9%) where ‘*x*’ is the log(SINV genome equivalents). The relative expression of host genes was determined by a ranked ΔΔ*C*t method in which the Δ*C*t was calculated by subtracting the *rpl8* *C*t value from the *C*t of the gene of interest. Then the Δ*C*t values were ranked to control for natural inter-individual variation in gene expression and the matching mock-infected Δ*C*t was subtracted from the treatment group Δ*C*t to obtain the ΔΔ*C*t. The fold-change in cDNA abundance was calculated by the equation 2^−ΔΔ*C*t^; see Tables S2–S7 for qPCR data presented in Figures 1–3, 5, and Figure S1. 

### 2.8. Statistical Analysis

All analyses were carried out using R v3.5.1 in RStudio v1.1.456 [90]. Unless otherwise stated, pairwise comparisons of gene expression were evaluated using the *pairwise.wilcox.test* function in the base R *stats* package (v3.6.2) to perform a Wilcoxon Rank Sums with a Benjamini–Hochberg correction for multiple comparisons. The correlation matrix was calculated and visualized using the *corrplot* package (v0.84) [91].

## 3. Results

### 3.1. Heat Treatment Reduces Viral Abundance in Adult Bees

To examine the impact of short duration temperature stress on the outcome of virus infection in honey bees, bees were infected via intrathoracic injection with the model virus Sindbis virus-GFP (SINV-GFP), allowed to recover for 6 h at 32 °C, exposed to heat shock (i.e., 42 °C for 4 h [88]), and then transferred back to 32 °C for the remainder of the study. Virus abundance in individual honey bees was assessed at 72 h post-infection by qPCR, and in three independent experiments heat shock significantly reduced SINV-GFP RNA equivalents (transcripts and genomes) (Figure 1). In replicate 1, heat-shocked bees had a 74% lower mean virus abundance (5.44 × 10^7^ ± 6.72 × 10^6^ SINV-GFP RNA copies) compared to bees maintained at a constant temperature (2.1 × 10^8^ ± 2.82 × 10^7^ SINV-GFP RNA copies; ANOVA, F = 27.16, *p* = 3.48 × 10^−5^). In replicate 2, heat-shocked bees had a 90% lower mean virus abundance (4.9 × 10^7^ ± 1.1 × 10^7^ SINV-GFP RNA copies) compared to bees maintained at a constant temperature (4.28 × 10^8^ ± 1.28 × 10^8^ RNA copies; ANOVA, F = 9.93, *p* = 0.0058). And, lastly, in replicate 3 heat-shocked bees had an 87% lower mean virus abundance (1.84 × 10^7^ ± 6.14 × 10^6^ SINV RNA copies) compared to bees maintained at a constant temperature (1.32 × 10^8^ ± 5.4 × 10^7^; ANOVA, F = 4.43, *p* = 0.048).

### 3.2. Virus Infection and Heat Shock Induce the Expression of Heat Shock Protein Encoding Genes

In order to identify the heat shock proteins (HSPs) potentially involved in reducing virus levels in heat-shocked honey bees, qPCR was used to measure the expression of six candidate HSP-encoding genes 72 h post-injection in virus-infected bees relative to mock-infected (buffer-injected) bees that were either maintained at a constant temperature or exposed to heat shock post-infection (Figure 2, Figure S1). The panel of honey bee heat shock protein encoding genes examined in these experiments included several genes that exhibited increased expression in previous transcriptome level analyses of virus-infected bees (i.e., *protein lethal(2)essential for life-like, dnaj shv-like,* and *hsp83-like*) [61], a gene involved in the unfolded protein response (i.e., *hsc70-3*), and core heat-shock response genes (i.e., *hsc70-4*, *hsp83-like*, and *hsp90*) [71].

*Protein lethal(2)essential for life-like (pl2)* is a small heat shock protein that contains an Hsp20-domain. Furthermore, *pl2* was the sole differentially expressed gene common to five transcriptome studies of virus-infected bees [14,48,49,61,67], including symptomatic bees that were orally infected with Israeli acute paralysis virus (IAPV) [48], naturally IAPV-infected bees [14], bees infected with deformed wing virus and sacbrood virus via oral inoculation [49], SINV-GFP-infected bees [61], and *Varroa destructor* parasitized and/or virus-infected bees [67].

In this study, *pl2* expression was greater in SINV-GFP infected bees compared to mock-infected bees in two of the three biological replicates (rep1, 1.55 fold, *p* = 5.1 × 10^−5^; rep2, 10.59 fold, *p* = 0.00013) but not in replicate 3 (*p* = 0.715) (Figure 2, Figure S1). Heat shock of mock-infected bees also resulted in greater *pl2* expression in two of three biological replicates (i.e., rep1, 2.86 fold, *p* = 5.1 × 10^−5^; rep2, 2.24 fold, *p* = 0.033), but decreased expression in replicate 3 (0.52 fold, *p* = 0.033) relative to mock-infected bees maintained at a constant temperature. For the majority of the samples, combining both stressors, virus infection and heat shock, induced *pl2* gene expression. Specifically, *pl2* expression in virus-infected heat-shocked bees was higher relative to mock-infected bees maintained at a constant temperature in two biological replicates (rep1, 4.93 fold, *p* = 0.00097; rep2, 40.5 fold, *p* = 0.00013), but not in replicate 3 (*p* = 0.715). Similarly, *pl2* expression in dual-stressed bees was greater than virus-infected bees maintained at a constant temperature in two of the replicates (rep1, *p* = 0.036; rep2, *p* = 0.00063), but not in replicate 3 (*p* = 0.715).

Previous transcriptome analyses of virus-infected honey bees determined that the expression of an Hsp40 gene *dnaj shv-like* was greater in virus-infected bees relative to mock-infected bees [61]. Likewise, in the experiments described herein, *dnaj shv-like* expression was greater in virus-infected bees relative to mock-infected bees (rep1, 1.39 fold, *p* = 9.2 × 10^−5^; rep2, 3.67 fold *p* = 0.00013; rep3, 3.74 fold, *p* = 5.4 × 10^−5^) (Figure 2, Figure S1). Heat shock also induced *dnaj shv-like* expression in mock-infected bees (rep1, 2.99 fold, *p* = 6.2 × 10^−5^; rep2, 1.39 fold, *p* = 0.00014; rep3, 2.81 fold, *p* = 5.4 × 10^−5^). Additionally, viral infection in conjunction with heat shock resulted in increased expression of *dnaj shv-like* relative to mock-infected bees maintained at a constant temperature (rep1, 1.46 fold, *p* = 0.018; rep2, 8.19 fold, *p* = 0.00013; rep3, 4.05 fold, *p* = 5.4 × 10^−5^). Combining stressors increased expression of *dnaj shv-like* over heat shock treatment alone in two replicates (rep2, *p* = 0.0074; rep3, *p* = 0.0028), in which virus-infected bees had an overall greater expression of this gene, and higher expression compared to heat-treated mock-infected bees (rep2, *p* = 0.00025; rep3, *p* = 0.0023). In contrast, mock-infected heat-shocked bees had greater *dnaj shv-like* expression in the first biological replicate.

*Heat shock 70-kDa protein cognate 3* (*hsc70-3*) is a conserved endoplasmic reticulum chaperone that functions in the unfolded protein response [71,92,93,94]. It is induced in honey bees exposed to heat stress (i.e., 45 °C for 4 h) [71]. In *Drosophila melanogaster* S2 cells *hsc70-3* is required for effective RNA-interference [78]. In the honey bee experiments described herein, SINV-GFP infection induced expression of *hsc70-3* over mock-infection in all three biological replicates (i.e., rep1, 1.76 fold, *p* = 6.8 × 10^−5^; rep2, 3.07 fold, *p* = 0.00013; rep3, 2.66 fold, *p* = 6.8 × 10^−5^). Though virus infection alone induced *hsc70-3* expression, heat shock alone did not result in increased expression in the majority of the bees and was only observed in one replicate (rep 1, 2.57 fold, *p* = 6.2 × 10^−5^). However, honey bees that were both virus-infected and heat-shocked had greater *hsc70-3* expression relative to mock-infected heat-shocked bees (rep1, 1.59 fold, *p* = 0.014; rep2, 3.25 fold, *p* = 0.00013; rep3, 2.90 fold, *p* = 6.8 × 10^−5^) (Figure 2, Figure S1).

*Heat shock 70-kDa protein cognate 4* (*hsc70-4*) is a core heat shock response gene that is induced by exposing honey bees to heat stress (i.e., 42 °C and 45 °C for 4 h) [71,95,96,97]. It is also an important chaperone for the assembly of the RNA-induced silencing complex (RISC) in *Drosophila* S2 cells and in flies [77,78]. Similar to previous studies, we determined that heat-shocked honey bees exhibited increased expression of *hsc70-4* relative to bees maintained at a constant temperature (mock-infected heat-shocked vs. mock-infected: rep1, 3.63 fold change, *p* = 5.1 × 10^−5^; rep2, 1.43 fold change, *p* = 0.00014; rep3, 2.44 fold change, *p* = 5.4 × 10^−5^). Virus infection also induced *hsc70-4* expression in two of the three replicates (rep2, 1.44 fold, *p* = 0.00013; rep3, 2.61 fold, *p* = 5.4 × 10^−5^). Combining stressors resulted in considerable heterogeneity in *hsc70-4* expression. In replicate 1, *hsc70-4* expression in bees that were virus-infected and heat-shocked was similar to mock-infected bees (*p* = 1.00) and significantly lower than bees exposed to heat shock alone (*p* = 4.4 × 10^−6^). In replicate 2, virus-infected bees that were heat shocked had 3.8 fold higher *hsc70-4* expression than mock-infected bees (*p* = 0.0013) and had higher expression than virus-infected bees (*p* = 0.0049) and bees that were only heat shocked (*p* = 0.0049). In replicate 3, bees that received both stressors had 2.28 fold higher expression of *hsc70-4* compared to mock-infected bees (*p* = 5.4 × 10^−5^), but no differences in expression compared to bees that were only virus-infected or only heat-shocked (Figure 2, Figure S1).

*Heat shock protein 83-like* (*hsp83-like),* a core heat shock response gene, exhibited increased expression in SINV-GFP infected bees [61,71,95,96]. It is also the homologue of *hsp83,* another chaperone of the RISC assembly in *Drosophila melanogaster* [77]. As expected, *hsp83-like* expression was greater in virus-infected bees than in mock-infected bees in three replicates (rep1, 1.20 fold, *p* = 0.038; rep2, 2.31 fold, *p* = 0.00012; rep3, 2.37 fold, *p* = 6.8 × 10^−5^). In contrast, *hsp83-like* expression was induced by heat shock in replicate 1, and specifically mock-infected heat-shocked bees exhibited 1.75 greater expression than mock-infected bees maintained at a constant temperature (*p* = 0.01), but had reduced expression in replicate 2 (0.27 fold change, *p* = 0.00012) and a trend toward reduced expression in replicate 3 (0.69 fold change, *p* = 0.05). However, reduced *hsp83-like* expression in virus-infected heat-shocked bees compared to virus-infected bees maintained at a constant temperature was observed in all three replicates (rep1, *p* = 0.0034; rep2, *p* = 0.00012; rep3, *p* = 0.00051) (Figure 2, Figure S1).

*Heat shock protein 90 (hsp90)*, another core heat-shock response gene, is induced in honey bees exposed to temperature stress (i.e., heat shock at 45 °C for 4 h) [71,95]. In this study, *hsp90* was consistently induced by viral infection (virus-infected vs. mock-infected bees, rep1, 1.24 fold, *p* = 0.01; rep2, 1.79 fold, *p* = 0.00012; rep3, 2.36 fold, *p* = 5.4 × 10^−5^). Heat shock resulted in higher *hsp90* expression relative to mock-infected bees in two of the three replicates (rep1, 3.59 fold, *p* = 5.1 × 10^−5^; rep3, 2.16 fold, *p* = 5.4 × 10^−5^), but expression was reduced in one replicate (rep2, 0.44 fold, *p* = 0.00012). Similar to the *hsc70-4* results, combining stressors resulted in considerable heterogeneity. *Hsp90* expression in honey bees that were both virus-infected and heat-shocked had mildly increased levels compared to mock-infected bees maintained at a constant temperature in replicate 1 (1.24 fold, *p* = 0.057), but had reduced expression in replicate 2 (0.68 fold, *p* = 0.00012) and increased expression in replicate 3 (2.55 fold, *p* = 5.4 × 10^−5^) (Figure 2, Figure S1).

### 3.3. Impact of Heat Shock on Expression of Honey Bee Antiviral Defense Genes

To discern whether the reduced virus abundance in heat-shocked honey bees was due to higher expression of honey bee immune genes, we assessed the relative expression of three honey bee antiviral defense genes (i.e., *mf116383, dcr-like*, and *ago2*, Figure 3) at 72 h post-infection [37,51,61]. These genes were identified in previous transcriptome level analyses that determined that their expression was greater in SINV-GFP infected honey bees [61]. Furthermore, the antiviral role of *dcr-like* and *mf116383* was confirmed in vivo [61]. Increased expression of *dcr-like* and *ago2* was also reported in transcriptome level analyses of IAPV-infected bees [48]. However, transcription of these key RNAi genes was not induced by deformed wing virus infection in honey bees [50] or virus infection of the model insect, *Drosophila melanogaster* [98,99,100,101]. *Mf116383* was not evaluated in these studies, as it was not well-annotated prior to 2017.

We identified and annotated the honey bee gene *mf116383* (GenBank) due to its increased expression in SINV-GFP infected bees relative to mock-infected bees and confirmed its role in limiting virus infection in honey bees [61]. Though the specific mechanism by which *mf116383* reduces virus infection is unknown, it was originally referred to as a probable cyclin-dependent serine/threonine-protein kinase based on prior partial annotation of LOC725387 (XM_001121241.4) [102,103,104]. In this study, *mf116383* was consistently increased in expression in virus-infected honey bees (rep1, 1.65 fold, *p* = 4.1 × 10^−5^; rep2, 4.76 fold, *p* = 0.00015; rep 3, 3.90 fold change, *p* = 6.8 × 10^−5^) (Figure 3A). *Mf116383* expression was also induced in mock-infected bees exposed to heat shock relative to mock-infected bees that were kept at a constant temperature (rep1, 1.86 fold, *p* = 4.1 × 10^−5^; rep2, 1.78 fold, *p* = 0.006; rep3, 2.66 fold, *p* = 0.0015). This is intriguing since virus-infected bees that were heat shocked harbored less virus than bees maintained at constant temperature post virus infection (Figure 1). Virus infection coupled with heat shock resulted in increased *mf116383* expression in two replicates relative to expression in mock-infected bees held at constant temperature (rep1, 3.5 fold, *p* = 4.1 × 10^−5^; rep3, 3.42 fold, *p* = 6.8 × 10^−5^), but showed no difference in replicate 2 (Figure 3A). However, a comparison of dual-stressed honey bees compared to mock-infected heat-shocked bees determined that *mf116383* expression was only appreciably greater in one replicate (rep1, *p* = 0.0012) and trended toward higher expression in another replicate (rep3, *p* = 0.056) (Figure 3A).

*Dcr-like* is an RNA helicase domain-containing endonuclease that recognizes dsRNA and processes it into short 21–22 nucleotide double-stranded short interfering RNAs (siRNAs) [45,105,106]. In this study, *dcr-like* expression was increased in virus-infected honey bees (rep1, 1.78 fold, *p* = 0.00032; rep2, 4.09 fold, *p* = 0.00013; rep3, 3.76 fold, *p* = 2.7 × 10^−5^) (Figure 3B). *Dcr-like* expression was also greater in mock-infected heat shocked honey bees compared to mock-infected bees maintained at constant temperature in two replicates (rep2, 2.11 fold, *p* = 0.00014; rep3, 1.35 fold, *p* = 2.7 × 10^−5^), but it was decreased in one replicate (rep1, 0.52 fold, *p* = 0.00032). Similarly, combining stressors resulted in increased expression compared to mock-infected bees in two replicates (rep2, 13.5 fold, *p* = 0.00013; rep3, 3.45 fold, *p* = 2.7 × 10^−5^), but not in replicate 1 (*p* = 0.115) (Figure 3B).

*Ago2* is the core endonuclease of the RISC, which when loaded with single-stranded siRNA targets complementary sequences in viral or cellular RNAs for cleavage [45,105,107]. As in previous studies, *ago2* expression was greater in virus-infected bees compared to mock-infected bees (rep1, 2.39 fold, *p* = 4.1 × 10^−5^; rep2, 4.0 fold, *p* = 0.00015; rep3, 4.18 fold, *p* = 2.7 × 10^−5^) (Figure 3C) [61]. Likewise, the expression of *ago2* was increased in virus-infected and heat-shocked bees compared to mock-infected bees held at constant temperature in two replicates (rep 1, 1.99 fold, *p* = 4.1 × 10^−5^; rep3, 5.13 fold, *p* = 2.7 × 10^−5^) (Figure 3C). However, *ago2* expression in heat-shocked mock-infected bees compared to mock-infected bees held at constant temperature was variable (i.e., rep1, 0.68 fold reduction, *p* = 4.1 × 10^−5^; rep2, no difference, *p* = 1.00; rep3, 2.23 fold increase, *p* = 2.7 × 10^−5^).

### 3.4. Honey Bee Immune Gene and Heat Shock Protein Gene Expression Positively Correlate

**Figure 4:**
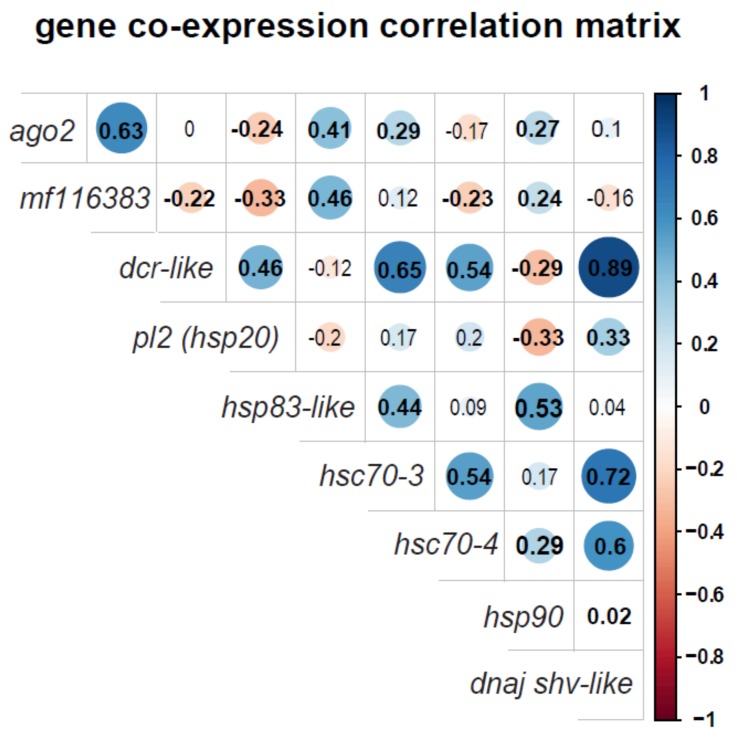
Honey bee gene co-expression correlation matrix. The co-expression of genes in virus-infected heat-shocked honey bees was analyzed by calculating the correlation coefficient for each pairwise comparison, which are listed in each cell. Correlation coefficients (*r*-values) quantify the strength and direction of the relationship between the expression of two genes. Shaded blue circles represent positive correlations while shaded red circles denote negative correlations. The larger the circle, the stronger the association and statistically significant correlations (*p* < 0.05) are indicated by a bold *r*-value. The matrix illustrates several significant positive correlations between immune genes (*dcr-like*, *ago2,* and *mf116383*) and heat shock proteins (*pl2, hsp83-like, hsc70-3, hsc70-4, hsp90, dnaj shv-like*), including *dcr-like* and *dnaj shv-like*, *hsc70-3* and *dnaj shv-like*, and *dcr-like* and *hsc70-3*.

Heat shock proteins play important roles in the insect antiviral response. Specifically, *hsp90* and *hsc70-4* act as chaperones for RISC assembly, thereby facilitating RNAi-mediated antiviral defense [77,78]. Therefore, to examine potential co-regulation of immune genes with HSP-encoding genes, a Pearson’s correlation coefficient matrix was calculated for each gene measured in this study, including data from four treatment groups (i.e., mock-infected, SINV-GFP-infected, mock-infected and heat shocked, and SINV-GFP-infected and heat shocked) with nine to twelve bees per biological replicate. In this analysis, *ago2* expression was positively correlated with the expression of three HSP-encoding genes, including *hsp83-like* (*r* = 0.41, *p* = 0.02), *hsc70-3* (*r* = 0.29, *p* < 0.0001) and *hsp90* (*r* = 0.27, *p* = 0.01) (Figure 4). *Dcr-like* expression was positively correlated with the expression of four HSP-encoding genes, including *pl2* (*r* = 0.46, *p* < 0.0001), *hsc70-3* (*r* = 0.65, *p* < 0.0001), *hsc70-4* (*r* = 0.54, *p* < 0.0001), and *dnaj shv-like* (*r* = 0.89, *p* < 0.0001). Lastly, *mf116383* expression positively correlated with the expression of two HSP-encoding genes, including *hsp83-like* (*r* = 0.46, *p* < 0.0001) and *hsp90* (*r* = 0.24, *p* = 0.02) (Figure 4 and Figure S2).

### 3.5. Increased Expression of Heat Shock Protein Encoding Genes Post Virus Infection Is Not Completely Recapitulated by dsRNA-Treatment

**Figure 5:**
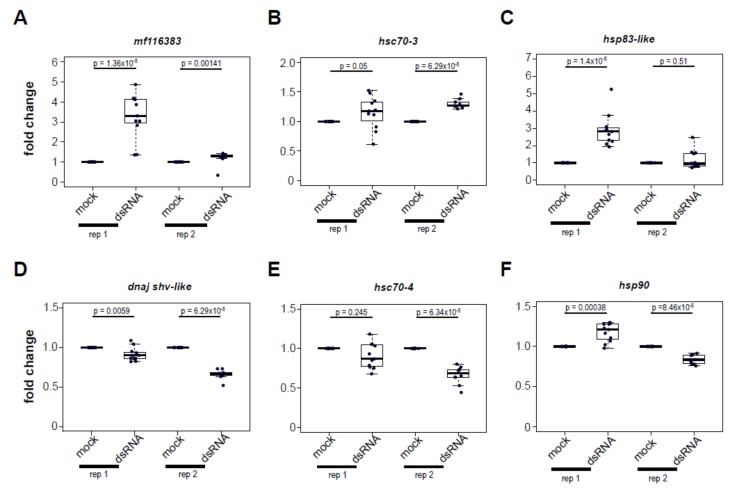
Treatment of honey bees with dsRNA, a virus-associated molecular pattern, differentially impacts heat shock protein encoding gene expression. To examine the impact of dsRNA, a virus-associated molecular pattern, on honey bee gene expression, bees were intrathoracically injected with dsRNA or buffer (*n* = 10–12 bees for each sample group). The relative expression of heat shock protein encoding genes was assessed by qPCR using the ΔΔ*C*t method with normalization to the internal control gene *rpl8* and relative to mock-infected bees and pairwise comparisons were analyzed by a Wilcoxon rank sums test. (**A**) *MF116383* expression was greater in dsRNA-treated honey bees for both replicates. (**B**) *Hsc70-3* expression was greater in dsRNA-treated bees in both biological replicates. (**C**) *Hsp83-like* in dsRNA treated bees was increased in expression in replicate 1, but not in replicate 2. (**D**) *Dnaj shv-like* expression was reduced in dsRNA-treated bees in both biological replicates. (**E**) *Hsc70-4* expression was reduced in dsRNA-treated bees replicate 2, but not in replicate 1. (**F**) *Hsp90* expression in dsRNA-treated bees was variable with greater expression in replicate 1 and lower expression in replicate 2.

To determine whether dsRNA, a virus-associated molecular pattern (VAMP), is necessary and sufficient to induce heat shock protein gene expression, honey bees were either injected with buffer or with buffer containing 1 μg of dsRNA, with sequence corresponding to the Drosophila C virus genome, and thus not specific to any honey bee gene or honey bee-infecting virus. As a control, we assessed the expression of *mf116383*, which is induced by dsRNA in a previous study [61], and, indeed, expression was induced at 72 h post dsRNA-injection (rep1, 3.29 fold, *p* = 1.36 × 10^−5^; rep2, 1.20 fold, *p* = 0.0014) (Figure 5). Likewise, the expression of five HSP-encoding genes 72 h post dsRNA-injection was measured by qPCR and the results varied for each gene assessed.

Specifically, dsRNA-treated bees exhibited greater expression of *hsc70-3* (rep1, 1.15 fold, *p* = 0.05; rep2, 1.30 fold, *p* = 6.29 × 10^−5^), and *hsp83-like* in replicate 1 (2.91 fold, *p* = 1.4 × 10^−5^), but not in replicate 2 (*p* = 0.51). In contrast, dsRNA-treated bees exhibited reduced expression of *dnaj shv-like* (rep1, 0.91 fold, *p* = 0.0059; rep2, 0.66 fold, *p* = 6.29 × 10^−5^) and *hsc70-4* in one of two replicates (rep2, 0.66 fold, *p* = 6.34 × 10^−5^). Injection with dsRNA had a variable impact on *hsp90* expression, increasing it 1.17-fold in the first replicate (*p* = 0.00038) and decreasing 0.83-fold in the second replicate (*p* = 8.46 × 10^−5^). Together, these results indicate that the HSP expression profile induced by virus infection is not completely recapitulated by exposure to dsRNA. Instead, there are likely other aspects of the virus-honey bee host interaction that result in differential regulation of genes in the heat shock stress response pathway.

## 4. Discussion

Honey bees have evolved a wide range of social and molecular strategies to control pathogens [37,38,39,51,108]. Viruses are associated with honey bee colony losses and individual mortality [14,20,22,24,25,26,27,47,48,49,50]. The outcomes of viral infections in individual honey bees are primarily governed by cellular immune responses, which include dsRNA-triggered immune responses (i.e., RNAi and non-sequence specific dsRNA mediated antiviral mechanisms) [14,46,48,50,59,60,61,109,110] and canonical immune signaling pathways, including the JAK/STAT, JNK, and Imd pathways, and the NF-κB/Dorsal mediated Toll pathway, which has been further characterized in vivo [20,111]. In addition, in several insect species behavioral fever reduces parasitic burden in individuals (reviewed in [112]). In honey bees, a group-level behavior termed “social fever” is hypothesized to function as a colony-level immune response. One study determined that colony temperature was increased in response to inoculation with fungal spores [40], although subsequent pathogen loads were not evaluated and the response was not consistently observed, perhaps due to environmental conditions [113]. Therefore, though “social fever” is an interesting aspect of honey bee biology, it requires further investigation and has not been observed in response to viral pathogens.

The mechanisms and genes involved in honey bee antiviral responses require further investigation. Herein, we present evidence that the heat shock response is involved in honey bee antiviral defense. First, we demonstrated that heat shock (i.e., exposure to 42 °C for 4 h) reduced the abundance of the model virus, SINV-GFP, compared to bees maintained at a constant temperature. One hypothesis that might explain the 74–90% reduction in virus abundance in heat-shocked honey bees is that there is a general disruption of protein synthesis in heat-stressed bees. However, drosophila cells completely recover normal protein synthesis upon return to physiologically normal temperatures after heat shock (four treatments at 37 °C for 25 min each) [114]. Furthermore, honey bee thoraces can reach in excess of 45 °C during foraging and aggression, suggesting they are adapted to cope with these high temperatures for short durations of time [115,116,117,118]. Therefore, disruption of protein synthesis is unlikely to be sufficient to explain the near log-reduction in viral abundance in heat-shocked bees at 72 h post-infection. Instead, it is likely that the transcriptional regulation of heat shock proteins is at least partially responsible for the antiviral effect of heat shock.

The expression of most of the heat shock protein encoding genes examined in this study was induced by virus infection in three biological replicates, except *protein lethal(2) essential for life-like* and *hsc70-4*, which were induced in two biological replicates. Though in general the expression of heat shock protein genes was induced by heat-treatment alone or with the combination of both stressors, there was some heterogeneity in expression in these treatment groups. These differences could be explained by either stochasticity, or by real differences in independent biological replicates for which we used three separate outbred honey bee colonies. These colonies include individual half-sisters of different genetic lineages that likely represent several different genetic sub-species prevalent in the U.S. [119]. Indeed, different honey bee subspecies (i.e., *A. mellifera carnica* and *A. mellifera ligustica*) have different thermotolerances and metabolic responses to heat stress [120]. Therefore, variation in honey bee genetic lineages could result in differential transcriptional regulation in response to heat shock. In addition, the biological replicates were carried out at different dates across two summers (i.e., June 2018, August 2018, and July 2019) when all the colonies were actively rearing brood, though there may have been variation in the physiological states of the colonies due to differences in weather and forage availability.

Virus infection induces the expression of numerous honey bee immune genes [14,46,48,49,61,67]. Similar to our previous study that examined the honey bee transcriptional response to SINV-GFP infection [61], we determined that virus infection resulted in increased expression of honey bee immune genes including *ago-2*, *dcr-like*, and *mf116383* [61]. However, *mf116383* was the only gene consistently induced by heat-treatment alone. Therefore, these data reveal a novel aspect of this recently described immune gene and suggests that *mf116383* may serve as one point of cross-talk between the generalized antiviral immune response and the heat shock response in honey bees. Since only ~35% of the honey bee genome has well-annotated orthologues with genes in other species including *Drosophila melanogaster*, there are numerous uncharacterized genes, like *mf116383*. It is exciting to further understand the biological role(s) of these genes in honey bees and other model and non-model organisms.

In contrast to the impact of heat shock on *mf116383* expression, heat shock had a variable effect on the expression of RNAi machinery across replicates. In some cases, the expression of *dcr-like* and *ago2* was reduced in heat shocked honey bees when compared to honey bees that were maintained at 32 °C. This implies that the mitigating effect of heat-treatment on virus infection is not simply explained by greater expression of the RNAi machinery. Instead, the protective effect of HSPs may be in part due to more efficient chaperone-mediated loading of the RISC [77,78]. Increased availability of chaperone proteins following heat shock may also ensure there are chaperones available for host-proteins as opposed to being occupied by viral proteins [79,80]. In addition to the expression of heat shock protein encoding genes correlating with each other, as expected, the expression of some HSP-encoding genes (e.g., *hsc70-3*, *hsc70-4,* and *hsp90*) was positively correlated with *dcr-like* and *ago2*. This suggests co-regulation of these genes. Though further studies are needed to determine the mechanisms leading to co-regulation of immune genes and HSP-encoding genes, it may be advantageous to co-regulate HSPs and HSP client proteins [77]. In addition to HSPs serving as chaperones for RNAi proteins, they may also play a direct antiviral role by interacting with viral proteins or may participate in, or mediate, broader stress-response pathways involved in antiviral defense.

The majority of viruses produce dsRNA molecules during their replication cycle (i.e., replicative intermediates of ssRNA viruses and secondary RNA structures, including internal ribosomal entry sites (IRES) and tRNA-like structures). Therefore, most host organisms have evolved mechanisms to detect dsRNA and subsequently trigger antiviral defense pathways. As expected based on a previous transcriptome analysis [61], *mf116383* expression was induced by dsRNA in the experiments described herein. Intriguingly, dsRNA-treatment did not fully recapitulate the HSP gene expression pattern that was observed in virus-infected bees. For example, *dnaj shv-like* and *hsc70-4* were both consistently induced by viral infection, but they had reduced expression in bees exposed to dsRNA alone. In contrast, the expression of both *hsc70-3* and *hsp83-like* was increased in virus-infected bees and dsRNA-treated bees. It is unclear which protein might be mediating transcriptional regulation of heat shock protein encoding genes in response to dsRNA, but it may be a protein like the mammalian dsRNA-dependent Protein kinase R (PKR), which is essential for the murine heat shock response and the expression of *hsp70* and *hsp84* [121]. Alternatively, an unidentified DEAD box helicase domain-containing protein, like those involved in cytosolic detection of dsRNA in mammalian cells (e.g., RIG-I, LGP2, MDA-5) may be mediating this response [122,123] (reviewed in [124]). There is some precedence for DEAD box helicase domain-containing proteins regulating gene expression. For example, the DEAD/H-box helicase Dicer-2 detects dsRNA and regulates the expression of the secreted antiviral peptide Vago in drosophila and mosquitoes [101,125,126]. Future studies will identify which honey bee protein or proteins are mediating differential expression of heat shock protein encoding genes in virus infection.

In summary, the work described herein indicates that stress response proteins, including those in the heat shock response and proteostasis network, are involved in honey bee antiviral defense. Further biochemical analyses are needed to confidently demonstrate their role in antiviral defense and the protective effect of heat shock. Future studies will aim to identify modes of coordination between stress and immune response pathways and proteins, as well as other potential antiviral functions of heat shock proteins, such as direct interaction with viral proteins.

## Supplementary Materials

The following are available online at https://www.mdpi.com/1999-4915/12/2/245/s1.
