# Supplementary material for: The Heat Shock Response in the Western Honey Bee (Apis mellifera) is Antiviral
Source: Viruses. 2020 Feb 22;12(2):245. doi: 10.3390/v12020245 (PMC7077298; doi:10.3390/v12020245)
Supplement: Supplementary file 1 [file viruses-12-00245-s001.zip › Combo Supplementary Materials/FigS3_pathogen screen.pdf]

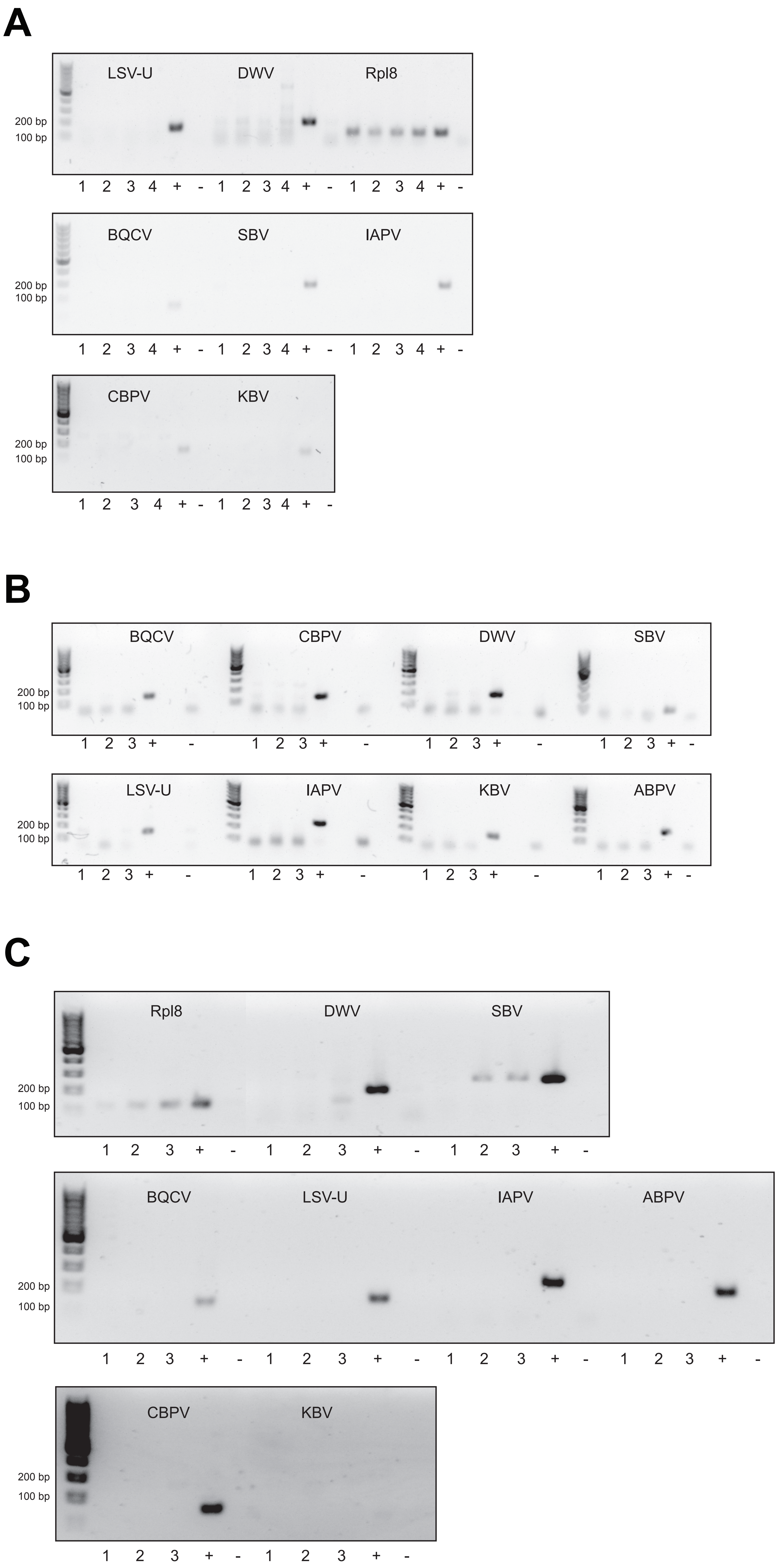

Supplementary Figure S3. Honey bee pathogen test results

Pathogen-specific PCR was used to test for presence of potential confounding, pre-existing infections in honey bees obtained from the same colony at the same time as the honey bees utilized in the experiments described herein. RNA was isolated from individual, untreated bees that were housed in conditions identical to the bees in the non-heat shocked treatment groups for the duration of the study. The quality of the cDNA was assessed using primers for the honey bee *rpl8* gene and the presence of the following viruses was assessed by PCR using primers listed in Supplementary Table S1 (i.e., LSV-U (Lake Sinai virus - universal), DWV (deformed wing virus), BQCV (black queen cell virus), SBV (sacbrood virus), IAPV (Israeli acute paralysis virus), CBPV (chronic bee paralysis virus), ABPV (acute bee paralysis virus) and KBV (Kashmir bee virus). +, positive control; -, negative control). Pathogen testing was performed on pooled cDNA samples representing four individual bees. PCR products were products were analyzed by agarose gel electrophoresis. (A) Pathogen test results of pooled cDNA samples from biological replicates 1 and 2 (Figures 1-4). Specifically, pools 1 and 2 are from rep1 bees, pool 3 consists of samples from reps 1 and 2, and pool 4 consists of rep2 cDNA samples. The PCR results for these samples indicate that the virus levels in biological replicates 1 and 2 were below the limit of detection. (B) Pathogen test results of three unique sets of pooled cDNA samples, representing 12 bees, from biological replicate 3 (Figures 1-4). The PCR results pooled cDNA samples 1, 2, and 3 indicate that the virus levels in biological replicate 3 were below the limit of detection. (C) Pathogen test results of three unique sets of pooled cDNA samples, representing 12 bees, from biological replicate 2 of Figure 5. The PCR results from pooled cDNA samples 1, 2, and 3 indicate that the virus levels were below the limit of detection for the majority of the samples, faint bands corresponding to SBV were detected in pools 2 and 3.
