# Supplementary material for: The Heat Shock Response in the Western Honey Bee (Apis mellifera) is Antiviral
Source: Viruses. 2020 Feb 22;12(2):245. doi: 10.3390/v12020245 (PMC7077298; doi:10.3390/v12020245)
Supplement: Supplementary file 1 [file viruses-12-00245-s001.zip › Combo Supplementary Materials/FigS2 CorrPlot pvalues.pdf]

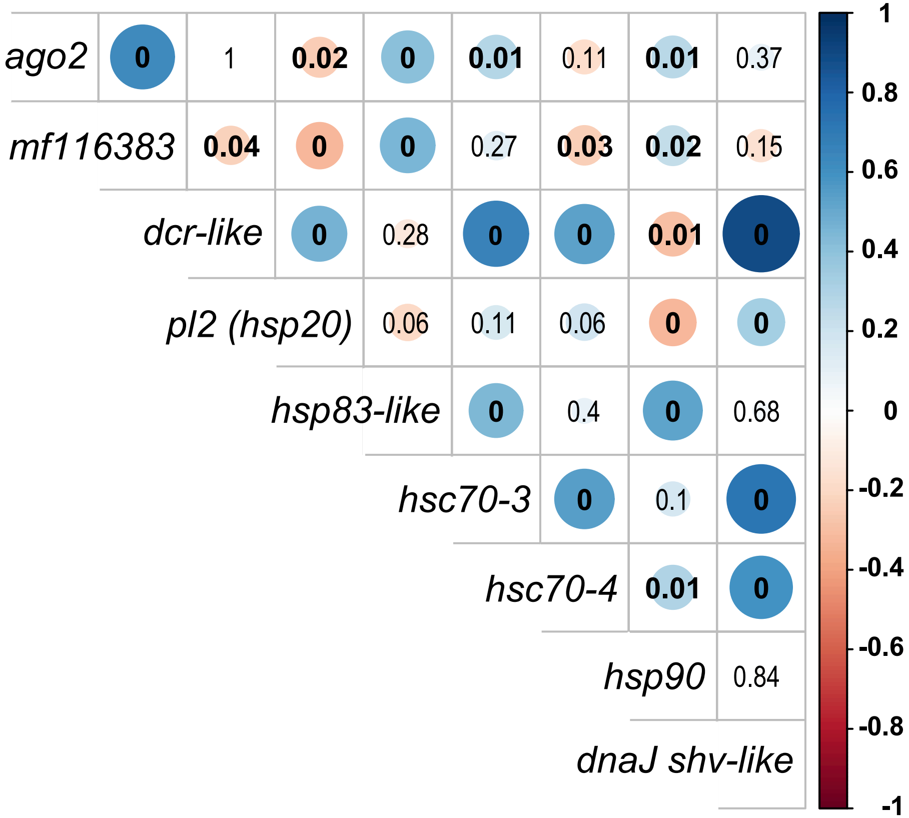

Supplementary Figure S2. Honey bee gene co-expression p-value matrix
The co-expression of genes in virus-infected heat shocked honey bees was analyzed by calculating the correlation coefficient for each pairwise comparison, each of which were assessed by calculating a corresponding p-value, which are shown in each cell. Correlation coefficients (r-values) are determined to be significant when their corresponding p-value is below 0.05. Shaded blue circles represent positive correlations while shaded red circles denote negative correlations. The larger the circle, the stronger the correlation. The matrix illustrates several significant positive correlations between immune genes (dcr-like, ago2, and mf116383) and heat shock proteins (pl2, hsp83-like, hsc70-3, hsc70-4, hsp90, dnaj shv-like), including dcr-like and dnaj shv-like, hsc70-3 and dnaj shv-like, and dcr-like and hsc70-3.
