# Supplementary material for: The Heat Shock Response in the Western Honey Bee (Apis mellifera) is Antiviral
Source: Viruses. 2020 Feb 22;12(2):245. doi: 10.3390/v12020245 (PMC7077298; doi:10.3390/v12020245)
Supplement: Supplementary file 1 [file viruses-12-00245-s001.zip › Combo Supplementary Materials/FigS1 Heat shock gene expression.pdf]

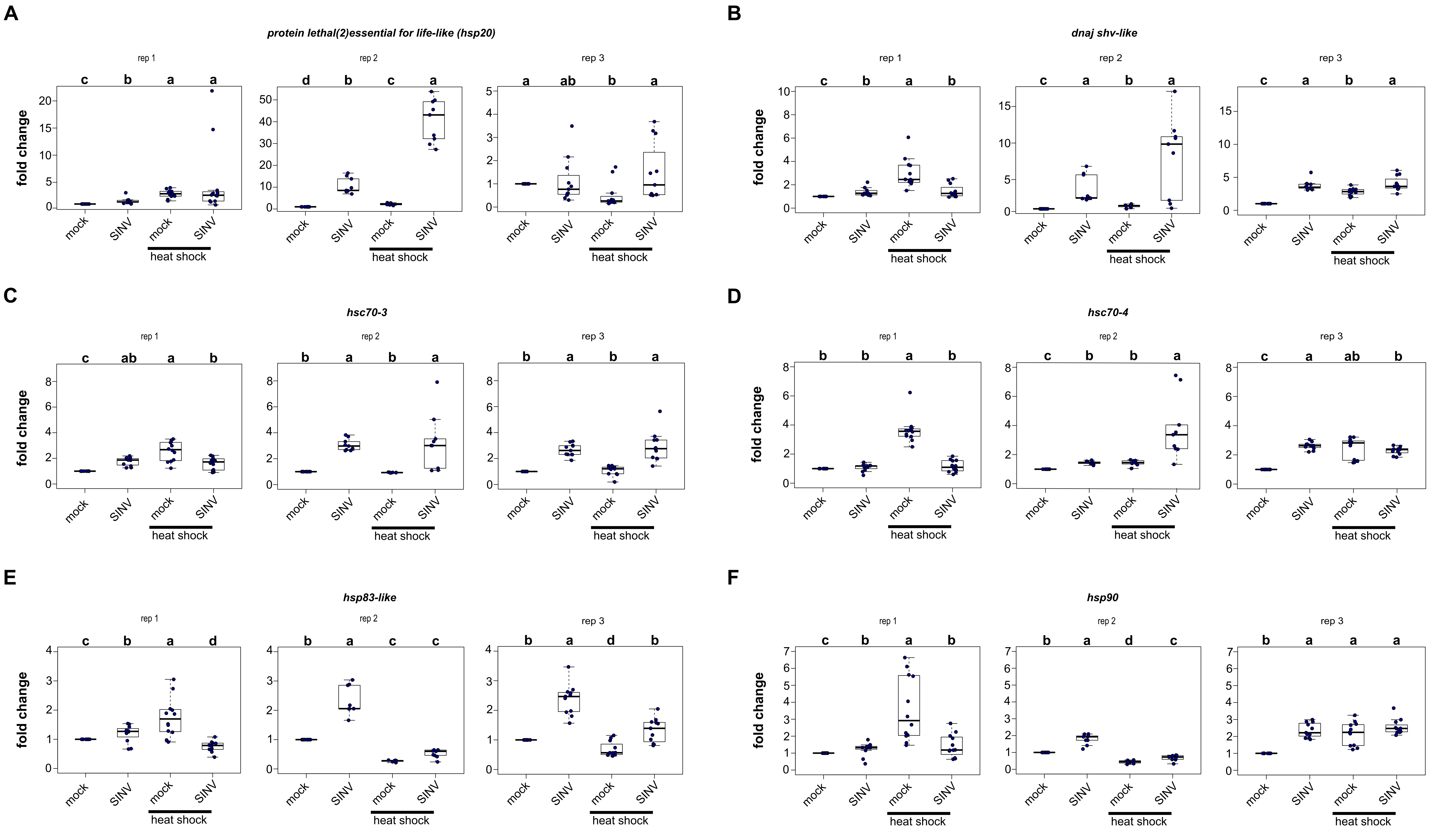

Supplementary Figure S1. Induction of heat shock proteins in 3 replicates

The relative expression of honey bee heat shock protein encoding genes was assessed by qPCR using the ΔΔCt method with normalization to rpl8 and relative to mock-infected bees maintained at a constant temperature. Unless otherwise stated, the following comparisons are relative to mock-infected honey bees.

(A) Protein lethal(2)essential for life-like (hsp20) was significantly increased in expression by both viral infection and heat shock in replicates 1 and 2, but not replicate 3. (B) Dnaj shv-like was significantly increased in expression by both viral infection and heat shock in all three replicates, including when the stressors were combined. (C) While heat shock 70-kDa protein cognate 3 (hsc70-3) expression was induced by viral infection in all three replicates, its expression was only increased by heat shock in replicate 1. However, hsc70-3 expression was increased in all three replicates where bees received both viral infection and heat shock. (D) Heat shock 70-kDa protein cognate 4, hsc70-4, expression was increased in heat shocked bees in all three replicates and viral infection induced this gene in replicates 2 and 3, but not in replicate 1. Likewise, combined heat shock and viral infection induced hsc70-4 expression in replicates 2 and 3, but not in replicate 1.

(E) Heat shock protein 83-like (hsp83-like) expression was induced by viral infection in all three replicates, while heat shock induced expression in replicate 1 but reduced its expression in both replicates 2 and 3. The combination of virus-infected and heat shock resulted in reduced hsp83-like expression relative to viral infection alone in all three replicates, indicating heat shock may suppress expression.

(F) Heat shock protein 90 (hsp90) expression was increased in virus-infected bees in all three replicates and mock-infected, heat shocked bees in replicates 1 and 3, whereas expression was reduced in replicate 2. Expression of hsp90 was induced by virus-infected, heat shocked bees in replicates 1 and 3, but its expression was reduced in replicate 2. Data were analyzed by a pairwise Wilcoxon Rank Sums with a Benjamini-Hochberg correction for multiple comparisons within replicates. Shared letters above two treatments denote there is no difference while different letters denote a statistical difference.
